# Exploring the causal effect of omega-3 polyunsaturated fatty acid levels on the risk of type 1 diabetes: a Mendelian randomization study

**DOI:** 10.3389/fgene.2024.1353081

**Published:** 2024-07-08

**Authors:** Lydia Abolo, Joachim Ssenkaali, Onan Mulumba, Olaitan I. Awe

**Affiliations:** ^1^ Department of Immunology and Molecular Biology, School of Biomedical Sciences, Makerere University, Kampala, Uganda; ^2^ Faculty of Medicine, Lira University, Lira, Uganda; ^3^ Department of Computer Science, University of Ibadan, Ibadan, Oyo, Nigeria; ^4^ African Society for Bioinformatics and Computational Biology, Cape Town, South Africa

**Keywords:** type 1 diabetes, omega-3 fatty acids, Mendelian randomization, causality, genetic variants

## Abstract

The burden of Type 1 diabetes (T1D) is vast and as of 2021, an estimated 8.4 million people were living with the disease worldwide. Predictably, this number could increase to 17.4 million people by 2040. Despite nearly a century of insulin therapy for the management of hyperglycemia in T1D, no therapies exist to treat its underlying etiopathology. Adequate dietary intake of omega-3 fatty acids (ω-3) has been reported in observational studies and Randomized Controlled Trials to be associated with reduced risk of developing T1D but results have been inconclusive. We conducted a Mendelian randomization (MR) study to explore the relationship between ω-3 intake and T1D. We performed a two-sample MR analysis using single nucleotide polymorphisms associated with ω-3 levels in a sample of 114,999 Europeans and their effects on T1D from a genome-wide association study meta-analysis of 24,840 European participants. A main MR analysis using the Inverse-variance weighted (IVW) method was conducted and validated using MR-Egger, Weighted median, and Weighted mode methods. Sensitivity analyses excluding potentially pleiotropic single nucleotide polymorphisms were also performed. Main MR analysis using the IVW method showed no evidence of a causal relationship between ω-3 levels and T1D risk (OR: 0.92, 95% CI: 0.56–1.51, *p* = 0.745). MR-Egger and Weighted mode methods showed similar results while Weighted median showed a marginally significant association (OR: 1.15, CI: 1.00–1.32, *p* = 0.048). Sensitivity analysis revealed heterogeneity in the main analysis MR estimates (IVW Q > 100, *p* < 0.0001) and no directional pleiotropy (Egger intercept: −0.032, *p* = 0.261). Our study found limited evidence of a causal association between ω-3 and T1D, with only a marginally significant association observed in one of the four MR methods. This challenges the proposition that ω-3-rich diets are of substantial benefit for the prevention and management of T1D.

## Introduction

Type 1 diabetes mellitus (T1D) is a chronic autoimmune disease characterized by the destruction of insulin-producing pancreatic beta cells and an ensuing lack of or low insulin (Bach, 1994; Eisenbarth, 2004). The burden of T1D is vast and as of 2021, an estimated 8.4 million people were living with the disease across the globe. It is predicted that by 2040, this number could increase to up to 17.4 million people (Gregory et al., 2022). Despite nearly a century of insulin therapy for alleviating hyperglycaemia and the accompanying symptoms of T1D, no therapies exist to treat the underlying etiopathology of the disease. Nonetheless, several large-scale trials have been conducted in recent years targeting interventions to preserve β-cell function and prevent or delay onset of their auto-destruction. Consortia such as the Type 1 Diabetes TrialNet (Bingley et al., 2018) and The Diabetes Prevention Trial--Type 1 Diabetes Study Group (2002) have embarked on coordinated efforts to develop disease-modifying interventions for at-risk individuals. One such trial from the T1D TrialNet involved the administration of oral insulin and found limited benefit of this intervention for preventing or delaying T1D onset. Additional studies have shown promising results for the use of immunotherapies that target immune cells or their pathways, as well as agents that induce immune tolerance to β-cells (Ke et al., 2020; Rapini et al., 2020; Rathod, 2022; Zhang et al., 2022) Other interventions such as islet transplantation and stem-cell therapy have also shown benefit in restoring insulin production, but these are limited by the short supply of donor islets and stem cells, and the risks associated with immunosuppression (Kort et al., 2011; Chen et al., 2020; Wan et al., 2022). Overall, these interventions present a promising outlook for T1D prevention but are hampered by their prohibitive costs (Tucker, 2022).

As evidenced by several studies, dietary interventions could offer a cost-effective approach to lowering the risk of T1D. Researchers have hypothesized that exclusive breastfeeding, delaying the introduction of cow’s milk and cereals, dietary intake of Vitamin D & E, zinc and polyunsaturated fatty acids (PUFA) are associated with T1D (Walter et al., 1991; Beales et al., 1994; Kimpimäki et al., 2001; Norris et al., 2003; Stene et al., 2003; Cardwell et al., 2012; Frederiksen et al., 2013). The role of fatty acid status in the development of T1D has been of notable interest to researchers. Particularly, adequate dietary intake of omega-3 fatty acids (ω-3) has been associated with a reduced risk of developing Diabetes (Baidal et al., 2016; Delpino et al., 2022; Elbarbary et al., 2023; Stene et al., 2003). A longitudinal, observational study, the Diabetes Autoimmunity Study in the Young (DAISY), conducted in 1,770 children at increased risk for T1D found that dietary intake of ω-3 is associated with reduced risk of islet autoimmunity (IA) in children (Norris et al., 2007). A metabolomic study by Niinistö et al. (2021) suggested that an altered early life fatty acid profile, which is somewhat linked with ω-3 intake, may predict risk for IA. While observational studies suggest an association between reduced ω-3 and increased risk of developing TID, a recent meta-analysis of randomized controlled trials (RCTs) established that ω-3 supplementation has limited benefit for the prevention of Type 2 diabetes in humans (Brown et al., 2019), and evidence for preventing T1D remains inconclusive and limited to animal studies (Bi et al., 2017).

The mechanisms by which ω-3 may have a protective effect against T1D are not fully understood. However, some studies have provided evidence for its potential role in allaying T1D through its anti-inflammatory effects (Delarue and Magnan, 2007; Newsholme et al., 2019; Poggioli et al., 2023). A recent study conducted in non-obese diabetic mice showed that mice that were fed on a PUFA-enriched diet had improved glucose tolerance, suggesting the possibility of an effect on β-cell function (Fenske et al., 2021). Some research has also suggested that ω-3 may help to regulate gut microbiota, which could have an impact on the development of autoimmune diseases such as T1D (Komaroff, 2017). The gut microbiome plays an important role in the immune system, and disturbances in its composition have been linked to the development of autoimmune diseases (Wu and Wu, 2012; Kaliannan et al., 2015).

Although RCTs are considered the gold standard for inference of causality, the long lead time between exposure (e.g., diet) and development of disease means that trials may take several years to produce robust results. Furthermore, the stringent inclusion criteria in RCTs limit generalizability in that conditions of the trial do not necessarily typify real life conditions. Mendelian randomization (MR) offers an alternative approach to inferring causality between exposures and outcomes (Sheehan et al., 2008; Davey Smith et al., 2020). These studies can be likened to a ‘natural’ RCT in that genetic factors are randomly assigned by nature at conception. MR exploits Mendel’s laws of segregation and independent assortment of alleles from parents to their offspring. As such, MR utilizes genetic variants related to an exposure of interest to proxy exposure variables with independence from confounding influences from other traits (Smith and Ebrahim, 2003). To the best of our knowledge, this approach has not been used to investigate a causal role for ω-3 deficiency in T1D. Given the scarcity of evidence from RCTs, we conducted an MR study using summary data from a GWAS of ω-3 polyunsaturated fatty acids (Borges et al., 2022) and a meta-analysis of 12 GWAS on T1D of individuals of European ancestry (Forgetta et al., 2020), to explore the relationship between ω-3 intake and T1D.

The findings from this study have significant implications for public health. As the global prevalence of T1D is predicted to increase over the next few decades, so will the economic burden associated with long-term management costs. The design and adoption of dietary and pharmacological interventions to alter the course of T1D in at-risk groups based on considerations of ω-3’s effects on T1D requires scientifically sound evidence. This study lends credence to these interventions in addition to opening new lines of inquiry for potential preventive and treatment strategies against T1D.

## Methods

**FIGURE 1:**
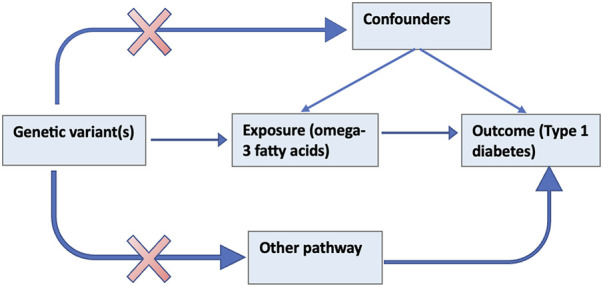
Schematic of the MR study design of the effect of ω-3 on T1D.

The MR method uses genetic variants that serve as a proxy for an environmentally modifiable exposure in order to make causal inferences about the outcome (Burgess and Thompson, 2017). Figure 1 below illustrates the theoretical underpinnings of the MR approach. The assumptions that must hold for a valid MR analysis are that; 1) the genetic elements chosen as proxies are robustly associated with the modifiable exposure (ω-3); 2) the genetic variants are not associated with confounders (e.g., BMI, vitamin D) that bias association between the modifiable risk and the outcome (referred to as horizontal pleiotropy) and; 3) the genetic variants influence the outcome only through the exposure (Smith and Ebrahim, 2003).

In this MR study, we used publicly available data obtained from the OpenGWAS database, a curated repository of complete GWAS summary datasets (Elsworth et al., 2020). A GWAS of ω-3 polyunsaturated fatty acids in a sample of 114,999 male and female European participants and a meta-analysis of 12 GWAS on T1D including a total of 9,358 cases and 15,482 controls of European ancestry. We identified SNPs associated with ω-3 and used these as proxies to explore the causal relationship between ω-3 and T1D.

### MR analysis workflow

**FIGURE 2:**
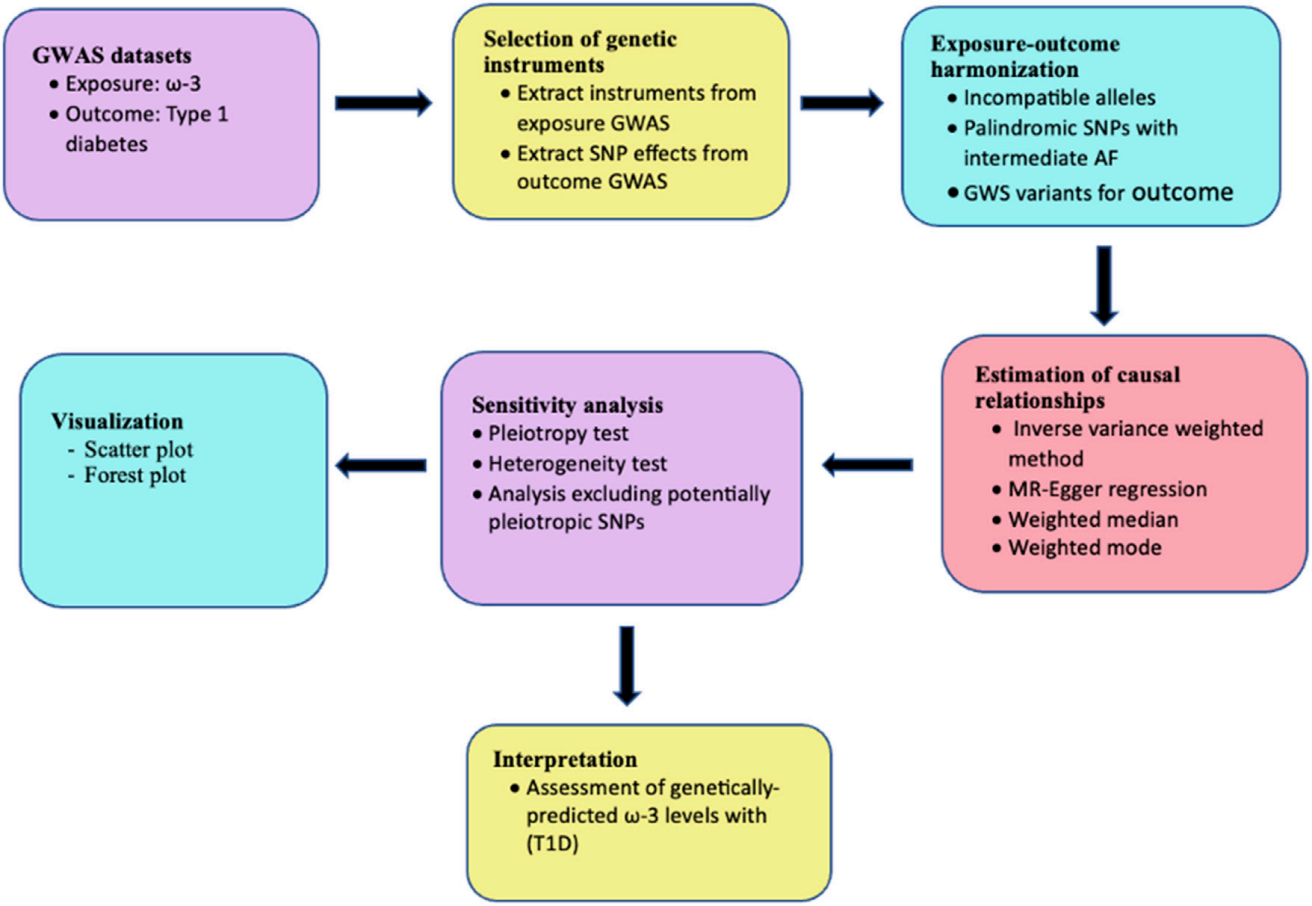
Analysis workflow for two-sample MR assessing the relationship between ω-3 and T1D.

This study was performed using a conventional two-sample MR design. The analysis was performed as shown in the workflow in Figure 2 and findings are reported based on the STrengthening the Reporting of OBservational studies in Epidemiology using Mendelian Randomization (STROBE-MR) guidelines (Skrivankova et al., 2021) (Supplementary Table S6). Ethical approval was not required for this study since the analysis was performed using publicly available data.

### Selection of genetic instruments associated with ω-3

To ensure that the instruments were robustly associated with the exposure of interest and conditionally independent, SNPs at a genome-wide significance level of *p* < 5e-8 were clumped at a distance of 10,000 kb and an r^2^ cut-off of 0.001. SNPs from the exposure dataset were then queried against the T1D GWAS summary statistics, and for those that were not measured in the outcome dataset, parameters allowed for proxy SNPs to be searched using the1000 Genomes European reference sample at a minimum linkage disequilibrium (LD) *r* ^2^ of 0.8. Palindromic SNPs were inferred at a minor allele frequency (MAF) threshold of 0.3. To obtain statistical evidence that the selected SNPs were sufficiently robust, the F-statistic was computed to measure the strength of the instrumental variables to predict the exposure using the formula β^2^ x (1–x), where β and x are the effect of the SNP on ω-3 and the MAF, respectively.

### Mendelian randomization analyses

Primary MR analysis was performed using Inverse-variance weighted (IVW) regression to estimate the genetically predicted effect of ω-3 levels on T1D susceptibility. The IVW method is considered the most accurate for the estimation of causal effects for two-sample MR analyses since it gives similar point estimates to an individual-level data analysis (Burgess et al., 2013). Specifically, the Wald ratio was used to estimate the effect of each instrumental variable on T1D risk weighted by its effect on ω-3 levels (Burgess et al., 2015; Boehm and Zhou, 2022). MR estimates of each instrumental variable were thereafter combined using a random effects inverse variance model. Additional MR analyses using MR-Egger, Weighted median, and Weighted mode methods were conducted to examine the validity of the results and provide more robust evidence for causal inference by confirming the consistency of results across different methods (Hemani et al., 2017). While MR-Egger regression estimates may be inaccurate if all IVs have similar magnitudes of association with the exposure, it can yield consistent estimates even when all selected IVs are invalid. The Weighted median method assumes that estimates from IVs without pleiotropic effects tend towards the median, whereas pleiotropy would be expected to introduce heterogeneity resulting in outliers. This method provides a precise causal estimate, even when up to 50% of the weight is from invalid IVs (Bowden et al., 2016). The weighted mode approach is less stringent on IV assumptions, granting pleiotropy even for the majority of the SNPs (Hartwig et al., 2017).

### Sensitivity analyses

In addition to a robust association between the IVs and the exposure, MR assumes that the genetic instruments affect the outcome only through the exposure and that they are not associated with traits that could bias the association between the exposure and outcome (Lawlor et al., 2008; Smith and Hemani, 2014). Violation of these assumptions is evidenced by the presence of heterogeneity and horizontal pleiotropy. Sensitivity analyses assessing for potential violation of these assumptions were conducted using the IVW and MR-Egger regression methods. Presence of heterogeneity was evaluated using the Cochran’s Q statistic and the existence of horizontal pleiotropy was evaluated using the Egger-intercept, considered at a significant *p*-value <0.05. Additionally, in order to identify IVs associated with potentially confounding GWAS traits, we queried the PhenoScanner database for each ω-3-associated SNP considering positive associations at a cut-off *p*-value of 5e-08 for genome-wide significant associations. This was done using the PhenoScanner package (Staley et al., 2016; Kamat et al., 2019) in R. The approach to analysing the PhenoScanner SNP-trait associations was adopted from a Mendelian randomization study by Manousaki et al. (2021) which investigated Vitamin-D levels and risk for T1D. SNP-trait associations from the PhenoScanner search were grouped into nine categories and those that did not belong to any of the categories were assigned to the ‘others’ category. SNP-trait associations occurring multiple times for each SNP from different GWASs were captured once (e.g., Total cholesterol, Cholesterol total and Cholesterol). The number of SNP-trait associations was determined for each category and five categories were selected to be included in the sensitivity analysis. Two categories with the highest number of SNP-trait associations (lipid- and blood-associated traits) were selected along with three categories chosen based on a biologically-plausible association (inflammation, body composition and Type 2 diabetes) that could confound the causal relationship between ω-3 and T1D. Sensitivity analyses were performed excluding SNPs associated with traits in each selected category respectively. The purpose for this exclusion was to examine any existing vertical pleiotropy in the relationship between the SNPs and ω-3. Further evaluation for horizontal pleiotropy was conducted using the Mendelian Randomization Pleiotropy RESidual Sum and Outlier (MR-PRESSO) method that detects horizontal pleiotropy (global test), corrects horizontal pleiotropy via outlier removal (outlier test) and performs testing of significant distortion in the causal estimates before and after outlier removal (distortion test) (Verbanck et al., 2018).

All MR analyses were implemented using the “TwoSampleMR” package (*version 0.5.6*) (Hemani et al., 2018) in R statistical software (*version 4.2.1*). The results of the analysis were presented as odds ratios (OR) with 95% confidence intervals (CI) and visualizations were presented using a scatter plot and forest plots. To test whether our study was sufficiently powered to estimate the causal effect of ω-3 on T1D risk, we used the method published by Brion et al. (2013), setting the alpha level at 0.05 and the variance in ω-3 levels explained by the IVs as calculated by the previously stated formula.

## Results

### Genetic instruments

After excluding SNPs in LD, clumped at a distance of 10,000 kb and an r^2^ cut-off of 0.001 using the 1,000 Genomes European reference panel, 52 SNPS that were significantly associated with the exposure and conditionally independent were identified as potential genetic instruments. 48 of the SNPs in the exposure instrument were available in the T1D GWAS data and proxies were found for two of the SNPs that were not available in the outcome dataset. Details on the specific SNPS used as genetic instruments including rs numbers, genomic positions, effect alleles, and their frequencies are captured in Supplementary Table S1. At the harmonization stage, seven palindromic SNPs and 1 SNP with incompatible alleles were removed leaving a total of 42 SNPs that were used as IVs in the main MR analysis. The variance in ω-3 levels explained by the genetic instruments was estimated at 10.2% and thus the IVs were robustly associated with the modifiable exposure.

### Mendelian randomization analysis

From our main MR analysis using the IVW method, there was no evidence of a causal relationship between ω-3 levels and risk of developing T1D (OR = 0.92 per 1SD increase in ω-3 levels, 95% confidence interval (CI): 0.56–1.51, *p* = 0.745). Further analyses using MR-Egger regression and Weighted mode methods similarly, did not reveal a causal association between the exposure of interest and the outcome (MR-Egger: OR = 1.20, CI: 0.61–2.35, *p* = 0.59, weighted mode: OR = 1.12, CI: 0.98–1.28, *p* = 0.10). However, analysis using the Weighted median method showed a marginally significant causal association between ω-3 levels and risk of developing T1D (OR = 1.15, CI: 1.00–1.32, *p* = 0.048).

Sensitivity analysis revealed evidence of heterogeneity in the MR estimates inferred from the 42 SNPs used in the main MR analysis (IVW Q > 100, *p* < 0.0001). There was no evidence of directional pleiotropy estimated across all IVs as deduced from the *p-*value of the Egger intercept (−0.032, *p* = 0.261). However, analysis using MR-PRESSO indicated presence of horizontal pleiotropy (global test *p*-value <0.001) and the outlier test identified four horizontal pleiotropic variants. Nonetheless, there was no significant distortion between the causal estimate before and after removal of the outlier variants (distortion coefficient = −26.23785, *p* = 0.456). The detailed results from the MR-PRESSO analysis are presented in Supplementary Table S2. MR analysis after removal of the two proxy SNPs gave similar results to those of the main MR analysis.

**FIGURE 3:**
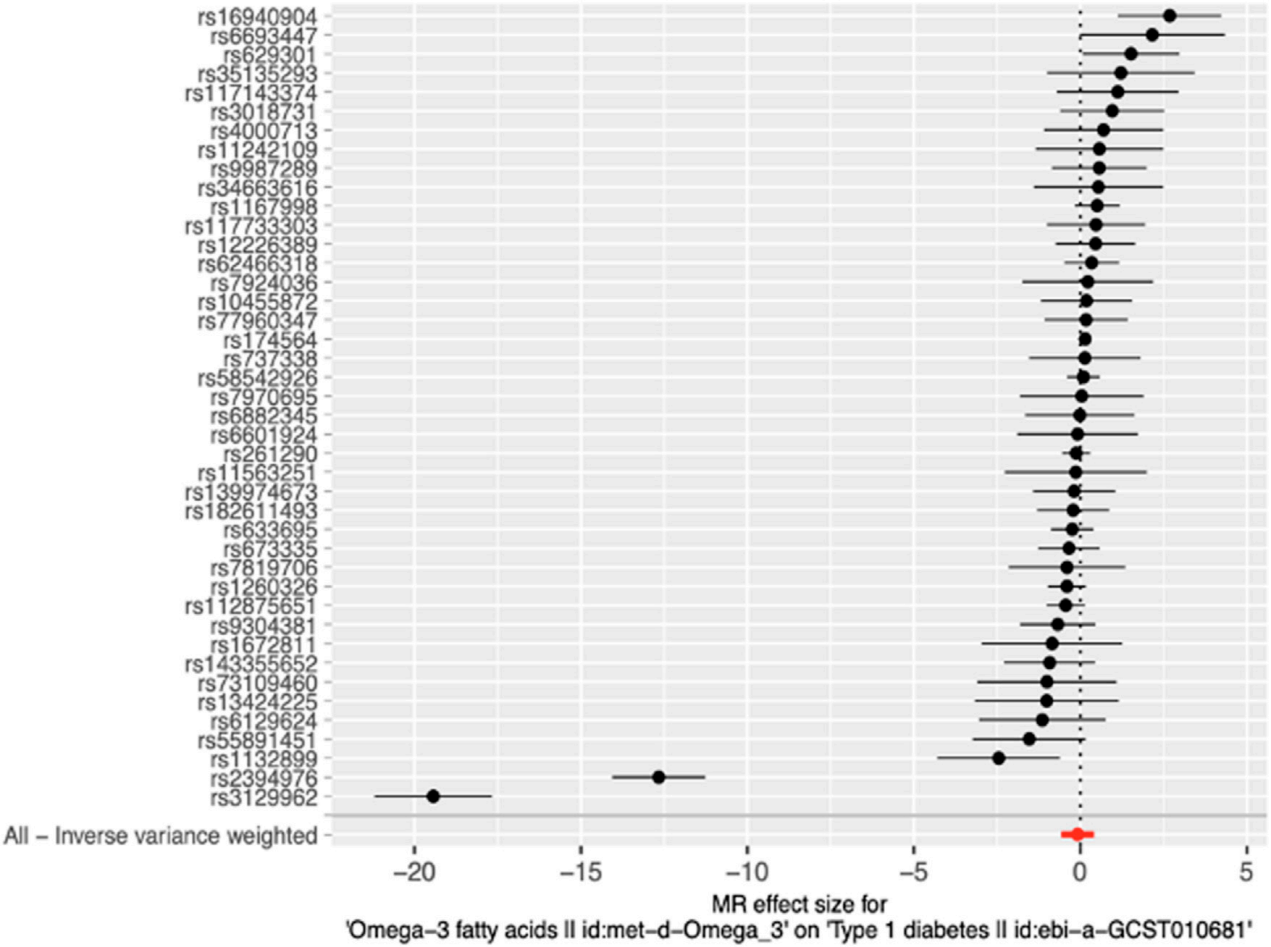
Forest plot of MR effect size for single SNP analysis and Inverse-variance weighted analysis on all SNPs. RSIDs for all instrumental variables are shown on the *y*-axis and their corresponding MR effect sizes estimated using the IVW method on the *x*-axis. The summary estimate for all IVs is represented as a red plotted point at the bottom of the graph. The vertical line through effect size of 0 represents the line of no effect.

**FIGURE 4:**
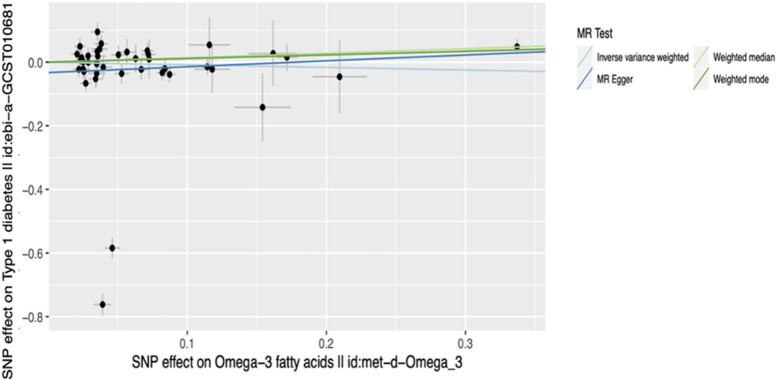
Scatter plot of main MR analysis. The horizontal axis represents the genetic correlation with omega-3 levels while the vertical axis represents the genetic association with T1D risk. Each coloured line on the graph denotes a distinct MR method.

**FIGURE 5:**
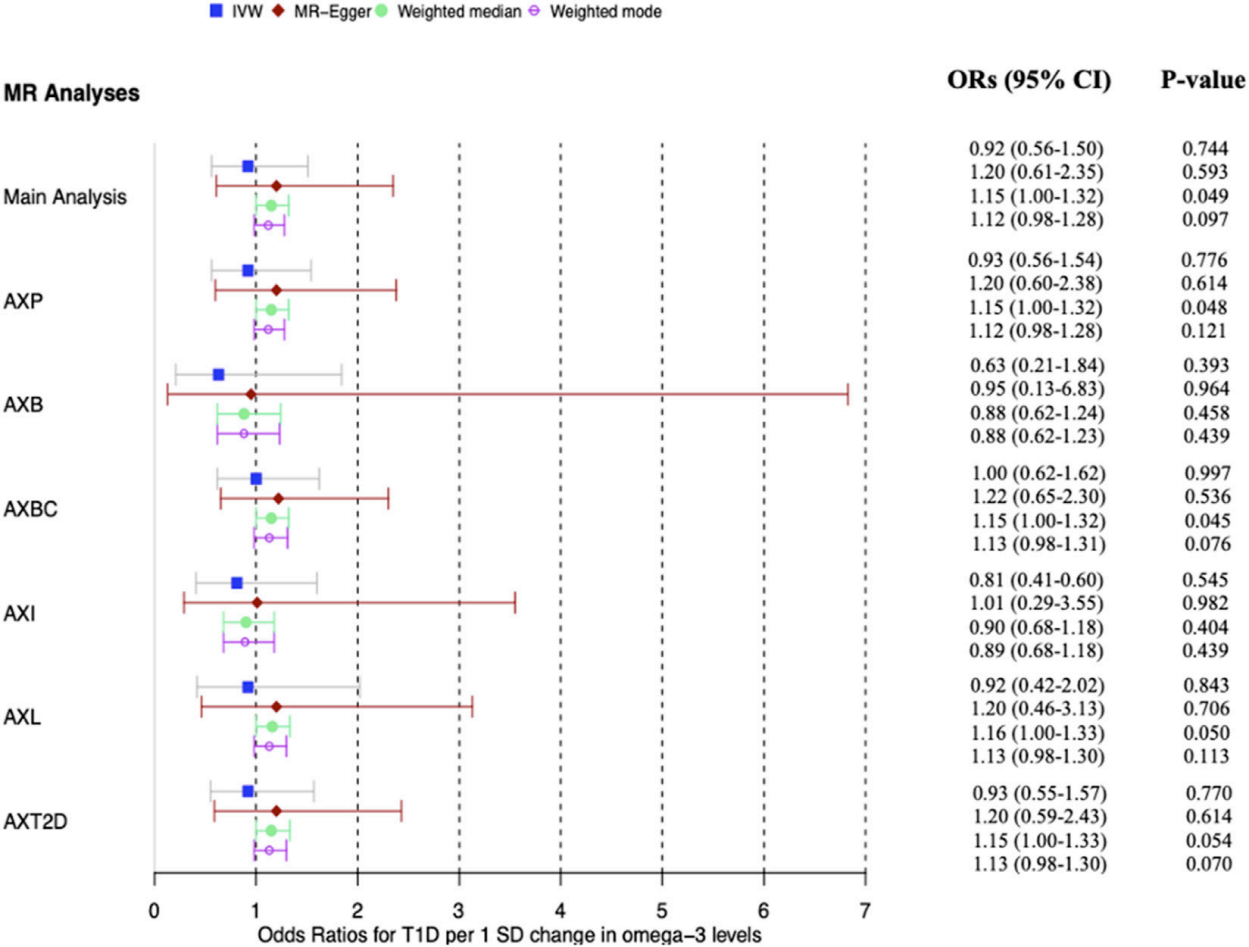
Forest plot of main MR analysis and sensitivity analyses excluding potentially pleiotropic variants. The odds ratios for T1D are reported for a 1 SD change in ω-3 levels. AXP: Analysis excluding proxy SNPs, AXB: Analysis excluding blood-associated SNPs, AXBC: Analysis excluding body composition-associated SNPs, AXI: Analysis excluding inflammation-associated SNPs, AXL: Analysis excluding lipid-associated SNPs, AXT2D: Analysis excluding Type 2 diabetes-associated SNPs, ORs: Odds ratios. The vertical grid line through OR one is the line of no effect.

Additional sensitivity analyses were conducted using output from the PhenoScanner search. Out of the 50 SNPs that were queried in the PhenoScanner database, 34 showed associations (1,131 associations) with 302 unique PhenoScanner traits (Supplementary Table S3). Analysis was performed using the IVs excluding those associated with lipid, blood, body composition, inflammation and T2D trait categories independently. Results similar to those from the main MR analysis, showing a marginally significant causal association for the Weighted median method, were obtained for the analyses which excluded SNPs associated with body composition, lipid and T2D trait categories. The analyses that excluded SNPs associated with blood and inflammation showed no significant causal effect of ω-3 levels on T1D risk. To determine whether the marginal significance of the causal effect estimated using the Weighted median method was attributable to SNPs associated with blood and inflammation traits, further MR analyses were performed using blood and inflammation-associated SNPs, independently. These revealed no significant causal effect for all methods, including Weighted median (blood: *p* = 0.057, inflammation: *p* = 0.051). Possible explanations for the disparity in the estimates of causal effects are that; 1) the assumptions of the different MR methods are not fully met; 2) the different MR methods vary in statistical power to detect causal effect, and; 3) the causal relationship between the exposure variable and outcome variable is very weak. All pleiotropy and heterogeneity tests yielded results similar to those of the main analysis. The detailed results of the main analysis and sensitivity analyses are presented in Supplementary Table S4 and can be visualized in Figures 3–5. Our study had the ability to detect the absence of effects on T1D with 80% power, given a sample size of 24,840 individuals, alpha level of 0.05%, and 10.2% variance explained by the IVs.

## Discussion

This MR study investigated the causal association between omega-3 polyunsaturated fatty acids and type 1 diabetes. The study aimed to provide evidence for the role of ω-3 in the prevention and management of T1D, a chronic disease with a growing incidence worldwide. The study approach utilized genetic variants associated with the exposure as proxies to establish a causal relationship with the outcome.

Our study found limited evidence of a causal association between ω-3 and T1D. The analysis showed that genetically predicted ω-3 levels were not significantly associated with the risk of T1D, with only a marginally significant association observed in one of the four MR analysis methods. Further interrogation of this observation by excluding proxy SNPs and potentially pleiotropic variants (SNPs associated with inflammation and blood-related traits) yielded no causal relationship inferred between the exposure and outcome. While the study had a large enough sample size and sufficient statistical power to detect meaningful associations, slight inconsistencies in the causal estimates across the MR methods, though not statistically significant, were observed. This could be due to unmeasured pleiotropic bias which could skew results away from the null and could not be ruled out by the sensitivity analyses that tested the robustness of the findings to different assumptions and variables. Nonetheless, due to the relatively wide confidence intervals in our results, it is possible that there are minor effects of ω-3 on the risk of developing T1D that we cannot dismiss.

These findings are consistent with those of previous observational studies and RCTs that showed no causal relationship between ω-3 status and T1D. A longitudinal study which followed 167 children with genetic predisposition to T1D for an average of 4.8 years concluded that ω-3 intake was not associated with conversion to T1D in children with islet autoimmunity (Miller et al., 2011). Relatedly, findings from a preliminary RCT of 20 participants, examining the efficacy of ω-3 for the treatment and management of T1D and its associated complications, revealed that ω-3 supplementation did not improve vascular health, glycaemic control, or metabolic parameters in subjects with T1D (O’Mahoney et al., 2020). However, these studies did not use the MR approach and currently no comparable studies have used this method to explore the omega-3-T1D link.

Our findings are contradictory to those from several observational studies that have suggested that higher ω-3 levels are associated with reduced risk of developing T1D (Norris et al., 2007; Bi et al., 2017; Cadario et al., 2017; Fenske et al., 2021). The possible mechanisms by which ω-3 may lower the risk of developing T1D are related to its anti-inflammatory effects. Inflammation is a predominant component of T1D, contributing to β-cell dysfunction and resultant cell death (Clark et al., 2017; Tsalamandris et al., 2019). Studies have suggested that ω-3 can help to regulate the immune system by reducing inflammation and promoting anti-inflammatory pathways, which may be beneficial in preventing or slowing the progression of T1D (Mori and Beilin, 2004; Calder, 2010; 2013). Another proposed mechanism is that ω-3 may have a direct effect on β-cells and other cells involved in glucose metabolism through regulation of gene expression (Delarue and Magnan, 2007; Newsholme et al., 2019). Whereas these studies have been instrumental in identifying a link between ω-3 status and diabetes, they are limited by potential confounding, selection bias and reverse causality (Sattar and Preiss, 2017; Hess and Abd-Elsayed, 2019; Nguyen et al., 2021). Reverse causation is possible in that having T1D could alter the metabolism of ω-3 or the absorption and utilization of these nutrients in the body, rather than the other way around. This could create a spurious association between ω-3 intake and T1D. Other factors that are associated with both ω-3 intake and T1D could also confound the observed relationship. For example, people who consume high levels of ω-3 may also have other healthy habits that reduce their risk of developing diabetes, such as exercising regularly or eating a nutrient-dense diet.

The MR approach used to investigate our hypothesis has various strengths. First, the use of genetic variants to proxy exposures in predicting disease risk is less prone to reverse causality as disease processes do not alter germline genotype. This is especially important for an outcome such as T1D for which the disease may have a preclinical stage that makes it hard to establish whether an exposure occurred before the underlying pathological changes. Secondly, genetic variations that are associated with a changeable environmental factor/exposure will remain linked to it from birth to adulthood. This implies that utilizing such genetic variations for causal inference can prevent the impact of errors due to regression dilution bias (Lawlor et al., 2008). Finally, the two-sample MR approach used in this study has the advantage of increased statistical power, particularly for testing causality on binary disease outcomes, because of the large sample size obtained from multiple GWAS (Davies et al., 2018). In this study, we used one of the largest T1D cohorts with 9,358 T1D cases and 15,482 controls, a sample size that is difficult to achieve for other study types.

In interpreting the results of our study, its potential limitations should be considered. Whereas we implemented rigorous steps to ensure the robustness of the genetic instruments used as instrumental variables, the stringent clumping distance of 10,000 kb and an r2 cutoff of 0.001 might have excluded SNPs that are close but independently associated with the exposure, potentially omitting relevant genetic variants from the analysis. Also, proxy SNPs could have introduced bias especially if these do not adequately represent the biological effect of the original SNPs not measured in the outcome dataset. Furthermore, although we used multiple genetic variants that collectively, were a strong genetic proxy for ω-3, and adequately satisfied the first MR assumption, we cannot completely rule out potential bias in our results due to unmeasured pleiotropy. Although we conducted several sensitivity analyses by excluding potentially pleiotropic SNPs and using methods such as MR-Egger, Weighted median and the mode-based estimator which are less sensitive to horizontal pleiotropy, residual bias cannot be definitively precluded. Potential bias in our results could also be introduced by canalization/developmental compensation (Waddington, 1942; Smith and Ebrahim, 2004). In the context of the relationship between ω-3 levels and T1D, canalization can occur through several mechanisms that may obscure the causal inference. One possible way is the presence of compensatory mechanisms that can mask the effects of ω-3 levels on the development of T1D. For example, it has been suggested that ω-3 may modulate immune function and inflammation, which could potentially reduce the risk of T1D. However, the immune system is highly complex, and it is possible that other compensatory mechanisms may be at play that can counteract the effects of low ω-3 levels, leading to a null result (Deem, 2005; Nish and Medzhitov, 2011; Paul, 2012). Another possible mechanism is the presence of genetic or epigenetic factors that can modulate the effects of ω-3 on the development of T1D. It has been proposed that the effects of ω-3 may depend on the individual’s genetic background or epigenetic modifications, which can affect the expression of genes involved in the regulation of immune function and inflammation (Hussey et al., 2017). If these factors are highly canalized, then the effects of ω-3 may be difficult to detect. To ensure homogeneity in our sample, we used GWAS data from individuals of European ancestry for both exposure and outcome datasets. Nonetheless, we acknowledge that potential confounding resulting from population stratification within this seemingly homogenous sample cannot be completely ruled out.

## Conclusion

Our findings challenge the proposition that ω-3-rich diets or supplementation is of substantial benefit for the prevention and management of T1D and its complications. While this study provides important information regarding the ω-3-T1D link, further evidence is required to explore the minor effects that ω-3 may have on T1D risk, as suggested by the marginally significant causal estimates detected by parts of our analysis. Extending this work by use of GWASs of larger sample sizes that may give more instruments and the use of multiple exposures to explore the extent to which our results and conclusions are likely to be robust would increase the precision of our findings. Finally, our deductions may need to be validated in non-European populations and by a robust RCT testing the influence of omega 3 on T1D risk.

## Data Availability

The original contributions presented in the study are included in the article/Supplementary Material, further inquiries can be directed to the corresponding author.
