# Supplementary material for: Exploring the causal effect of omega-3 polyunsaturated fatty acid levels on the risk of type 1 diabetes: a Mendelian randomization study
Source: Front Genet. 2024 Jul 8;15:1353081. doi: 10.3389/fgene.2024.1353081 (PMC11260775; doi:10.3389/fgene.2024.1353081)
Supplement: Supplementary file 2 [file Table1.DOCX]

**Supplementary Table 4**: Results of the MR and sensitivity analyses evaluating causal relationship between omega-3 fatty acids and type 1 diabetes.

| **MR ANALYSES** | **ODDS RATIO** | **LOWER CI** | **UPPER**  **CI** | **EGGER INTERCEPT (p.value)** | **HETEROGENEITY (Cochran's Q,**  **p.value)** |
| --- | --- | --- | --- | --- | --- |
| **ALL SNPS (N=42)** | | | | | |
| Inverse variance weighted | 0.921592283 | 0.563837 | 1.506343017 |  |  |
| MR Egger | 1.202422389 | 0.61481 | 2.351654213 | -0.03284337 (0.261) | 807.4 (p<0.0001) |
| Weighted median | 1.151203889 | 1.001443 | 1.323360399 |  |  |
| Weighted mode | 1.121528134 | 0.981042 | 1.282132241 |  |  |
| **Analysis excluding proxies (N=40)** | | | | | |
| Inverse variance weighted | 0.929514828 | 0.561293 | 1.539298121 |  |  |
| MR Egger | 1.196317331 | 0.600105 | 2.384874093 | -0.0316112 (0.301) | 806.3 (p<0.0001) |
| Weighted median | 1.151346635 | 1.001571 | 1.323519325 |  |  |
| Weighted mode | 1.119989134 | 0.981791 | 1.277639731 |  |  |
| **Analysis excluding blood-associated snps (N=25)** | | | | | |
| Inverse variance weighted | 0.6257817 | 0.2124 | 1.843482 |  |  |
| MR Egger | 0.9548689 | 0.1334 | 6.832917 | -0.02624965 (0.617) | 479.4 (p<0.0001) |
| Weighted median | 0.8778689 | 0.6239 | 1.235306 |  |  |
| Weighted mode | 0.8764461 | 0.6245 | 1.230132 |  |  |
| **Analysis excluding body composition -associated snps (N=32)** | | | | | |
| Inverse variance weighted | 1.001497 | 0.6185 | 1.621708 |  |  |
| MR Egger | 1.22347 | 0.6499 | 2.303358 | -0.02762246 (0.345) | 490.0 (p<0.0001) |
| Weighted median | 1.153511 | 1.0044 | 1.324776 |  |  |
| Weighted mode | 1.132829 | 0.9825 | 1.306164 |  |  |
| **Analysis excluding inflammation-associated snps (N=36)** | | | | | |
| Inverse variance weighted | 0.8111339 | 0.4107 | 1.601869 |  |  |
| MR Egger | 1.0129763 | 0.2894 | 3.545897 | -0.01514672 (0.680) | 507.3 (p<0.0001) |
| Weighted median | 0.8951523 | 0.6769 | 1.183707 |  |  |
| Weighted mode | 0.8940344 | 0.6756 | 1.183157 |  |  |
| **Analysis excluding lipid-associated snps (N=24)** | | | | | |
| Inverse variance weighted | 0.9249223 | 0.4238 | 2.018688 |  |  |
| MR Egger | 1.203913 | 0.4629 | 3.130824 | -0.04304142 (0.357) | 779.6 (p<0.0001) |
| Weighted median | 1.1551229 | 1.0042 | 1.328662 |  |  |
| Weighted mode | 1.1265535 | 0.9771 | 1.298821 |  |  |
| **Analysis excluding T2D-associated snps (N=40)** | | | | | |
| Inverse variance weighted | 0.92505 | 0.5467 | 1.56524 |  |  |
| MR Egger | 1.200237 | 0.5931 | 2.428725 | -0.03221034 (0.285) | 806.6 (p<0.001) |
| Weighted median | 1.153055 | 1.0001 | 1.329377 |  |  |
| Weighted mode | 1.130542 | 0.9835 | 1.299632 |  |  |
